# Engineered and Mimicked Extracellular Nanovesicles for Therapeutic Delivery

**DOI:** 10.3390/nano14070639

**Published:** 2024-04-06

**Authors:** Verena Poinsot, Nathalie Pizzinat, Varravaddheay Ong-Meang

**Affiliations:** Inserm, CNRS, Faculté de Santé, Université Toulouse III—Paul Sabatier, I2MC U1297, 31432 Toulouse, France; nathalie.pizzinat@inserm.fr (N.P.); varravaddheay.ong-meang@inserm.fr (V.O.-M.)

**Keywords:** extracellular vesicles, exosomes, hybrid exosome, exosome-mime, drug delivery

## Abstract

Exosomes are spherical extracellular nanovesicles with an endosomal origin and unilamellar lipid-bilayer structure with sizes ranging from 30 to 100 nm. They contain a large range of proteins, lipids, and nucleic acid species, depending on the state and origin of the extracellular vesicle (EV)-secreting cell. EVs’ function is to encapsulate part of the EV-producing cell content, to transport it through biological fluids to a targeted recipient, and to deliver their cargos specifically within the aimed recipient cells. Therefore, exosomes are considered to be potential biological drug-delivery systems that can stably deliver their cargo into targeted cells. Various cell-derived exosomes are produced for medical issues, but their use for therapeutic purposes still faces several problems. Some of these difficulties can be avoided by resorting to hemisynthetic approaches. We highlight here the uses of alternative exosome-mimes involving cell-membrane coatings on artificial nanocarriers or the hybridization between exosomes and liposomes. We also detail the drug-loading strategies deployed to make them drug-carrier systems and summarize the ongoing clinical trials involving exosomes or exosome-like structures. Finally, we summarize the open questions before considering exosome-like disposals for confident therapeutic delivery.

## 1. Introduction

Besides the apoptotic bodies, endogenous cell-membrane-based vesicles are secreted by most cell types and are called extracellular vesicles (EVs). EVs adopt various shapes and sizes, from one micrometer to several nanometers. Described for the first time about 30 years ago [1], exosomes are the nano-sized members of this vast family. These are spherical nanovesicles with an endosomal origin and unilamellar lipid bilayer structure [2] with sizes ranging from 30 to 100 nm. Exosomes were first identified as garbage bags [3], but their capability to stimulate adaptive immune responses has been rapidly observed [4]. The biogenesis isolation and characterization methods have been widely reviewed (e.g., [5,6]).

EVs were found to contain a large range of proteins, lipids, and nucleic acids species [7,8,9] in their aqueous core, but also membrane proteins, MHC (major histocompatibility complex) molecules, and integrins embedded in the phospholipid membrane. Moreover, their content depends on the state and origin of the EV-secreting cell [10]. Consequently, EVs are demonstrated to be heterogeneous in both content and functionality [11,12]. The EVs’ function is to encapsulate part of the EV-producing cell content, to transport it through biological fluids, and to a targeted recipient. Indeed, exosomes were demonstrated to be able to target (even at distance) cells that are different from parent cells (usually called recipient cells). After targeting, these nanovesicles are internalized to deliver their cargos specifically within the recipient cells. This fate implies excellent biocompatibility, low toxicity, and immunogenicity. It produced an increasing interest in exploiting EVs for diagnostic and therapeutic purposes, and even clinical trials [13,14,15,16,17]. It can be noticed that, until the pure spherical nanosizing of the exosomes cannot be proved, exosomes should be called EVs or nanosized EVs [18].

In line with their vesicular structure, EVs are permeable and quite dynamic systems. However, the knowledge on how EVs can transfer their content to recipient cells remains limited but central for the further development of EV-inspired therapies. The review of Kooijmans et al. [19] gives the landscape and latest insights in critical effectors in the EV–cell interface.

**Figure 1:**
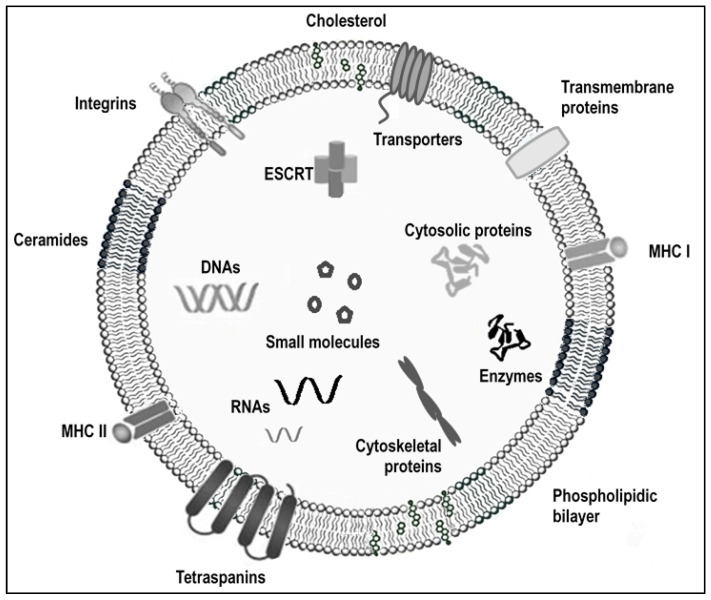
Schematic representation of the composition (families of proteins, lipids, and nucleic acids) and membrane orientation of EVs. Tetraspanins are commonly found in EVs and include CD63, CD81, and CD9. Each listed component may be present but not always simultaneously. Abbreviations: ESCRT, endosomal sorting complex required for transport; MHC, major histocompatibility complex. This figure is inspired by the one published by Colombo et al. [2].

Structurally, EVs are biological members of the lipidic nanoparticles family, as their formation involves all lipid-type pathways, lipid molecular types, and lipid transporter metabolism [20]. Lipids—with approximately 280 molecular species—constitute the second component in the exosome membrane [21]. Lipids and lipid rafts are also one of the main components of exosomes obtained from donor cells [22,23]. Even if exosomes are formed by the endosome’s membrane invagination, the lipid composition of exosomes differs when compared to the parent-cell membrane. Exosomes contain the CD36 in their cargo, which acts as fatty acid translocase [24], allowing individual nutrition and exogenous lipids to modify their lipid structure. Various factors influence the abundance and composition of these lipids, including the producer-cell line, cell type, and the physiological stage of the parent cell [25] (Figure 1).

The lipidomics array demonstrated this parent-cell specificity, as ceramide and gangliosides are the dominant lipids in exosomes derived from neuronal and glial cells [26]. In contrast, cholesterol, sphingomyelin, and ceramides are enriched in exosomes deriving from mouse neuroblastoma [27]. Endosomal sorting complex required for transport (ESCRT)-dependent and -independent mechanisms are proposed to explain this statement. ESCRT is composed of four complexes [28]. The ESCRT-0 complex recognizes and sequesters ubiquitinated transmembrane proteins in the endosomal membrane. The ESCRT-I and -II complexes are implicated in membrane-bud formation with sorted cargos. ESCRT-III drives vesicle ablation [29]. Therefore, ESCRT-I and -II determine the lipid content of exosomes, but the lipid raft-mediated cargo loading is ESCRT-independent. These domains are enriched in cholesterol, sphingolipids, and GPI-anchored proteins that are involved in the protein sorting [30]. Phospholipase D2 [31] and diacylglycerol kinase [32] are involved in the maturation of endosomes, especially the formation of the intra-lumiminal vesicles [33]. The sphingomyelinase and bis-monoacylglycerol phosphate play key roles in exosome biogenesis, as well as budding and release [34]. An increased amount of cell ether lipids also facilitates exosome release by increasing the late endosome fusion with the plasma membrane. Finally, the phosphoinositides, PIP3, PI (3,5), and P2 also help the formation of exosomes by inhibiting the PI3K/Akt (a negative regulator of the exosome secretion signaling pathway) [35,36]. The amount of cholesterol in the late endosomes is decisive in their fate. Indeed, cholesterol-rich vesicles fuse with the cell plasma membrane and its content released into the extracellular space, while cholesterol-free vesicles are directed to the lysosome for destruction [31].

Exosomes are involved in lipid synthesis, transportation, and degradation [37]. Referring to the proteins, the amount of lipids in exosomes is four times higher compared to the original cells content [38]. Moreover, their lipid composition differs from the one of the parent cell [21,39]. In accordance with their formation and release mechanisms, the exosomes are globally enriched with sphingomyelins, phosphatidylserins, gangliosides, glycosphingolipids, phosphatidic acid, phosphatidylcholins, phosphatidylethanolamins, and cholesterol [21,40]. However, these were reported to be impoverished in diacylglycerol, relative to the membranes of their cells of origin [41,42,43].

In addition to their lipidic structure, exosomes are biological vehicles that directly transfer lipids (including arachidonic acid and eicosanoid) [44] and contain various lipid transporters. Exosomes also contain a variety of enzymes involved in lipid metabolism, including phospholipases D, A2 [45], and oxilipin synthases (i.e., 5- and 15-lipoxygenase, leukotriene A4 hydrolase, and leukotriene C4 synthase, PGD synthase) [46,47].

Exosomes significantly impact the immune system. Indeed, tumor cells actively release exosomes that interplay with the immune system and the cancer development itself. Immunosuppressive molecules present in tumor-derived exosomes enable cancer cells to evade the immune system by impairing the function of immune cells such as the cytotoxic T lymphocytes or NK cells, allowing them to spread and proliferate. Conversely, many tumor-derived exosomes contain tumoral antigens that may be delivered to dendritic cells and stimulate T-cell-mediated antitumor immune response [48]. Similarly, innate immune cell-derived exosomes take part in antitumor immune responses and their properties could be enhanced to target and eliminate cancer cells [49]. Moreover, several vaccine studies have demonstrated that infected cells secrete exosomes containing multiple antigens from the hosted pathogen, helping to overcome pathogen escape from the immune system [50].

Liposomes are lipidic nanoparticles (LNPs) with spherical hollows and a lipid bilayer structure, allowing to encapsulate a variety of drugs within the lipid membrane or into the cavity (e.g., [51,52,53,54]). Even if they demonstrate good biological compatibility, increasing their stability, retention time in blood, and the efficiency of the drug delivery was necessary [55,56,57,58]. The use of PEGylated lipids, pH-sensitive cationic lipids, enabled their evaluation in a clinical trial [59]. 

LNPs and EVs allow two different targeting modes: the active and passive ones [60,61,62]. Active targeting can be achieved for the liposomes by conjugating specific ligands to the LNP surface, while EVs are biosynthesized with a large toolbox allowing them to bind to specific target cell receptors, mediating their inclusion. Although LNPs offer many advantages for drug-delivery systems, their poor stability in circulation causes drug release in non-target organs [63]. Therefore, exosomes are considered as drug-delivery systems, biologically produced, that can stably deliver their cargo into targeted cells.

Various cell types have been used to produce exosomes for therapeutic issues, including cancer model cell lines (e.g., [64,65]) and immature dendritic cells [66]. Unfortunately, their use for therapeutic purposes faces several problems. First, for all types of investigated cells, a scaling-up of the exosome production yield is required for clinical translatability [67,68]. Until now, mesenchymal stem cells (MSC) are the quantitatively best exosome-producing cells [69]. Secondly, their biogenesis and secretion are fine-regulated processes affected by numerous factors, like pH, calcium concentration, and cellular state [7]. Consequently, their production remains unreproducible in amount and composition. Finally, due to their size, density, and presence of membrane proteins, their isolation from the free proteins (with potentially undesired immunogenicity or activity) of the surroundings is a real challenge. Therefore, nanosized-EVs cannot be easily used in clinical trials as high-performance drug carriers, and developing synthetic exosome mimes or engineered biological nanoEVs remains a suitable strategy. 

In this review, we first summarize the ongoing clinical trials involving exosomes. Then, we report on therapeutic assays involving engineered exosomes, including their biological modifications and the drug-loading strategies used to transform exosomes into therapeutic carriers. We also highlight the uses of the alternative exosome mimes including the cell-membrane coating on artificial nanocarriers and the hybridization between exosomes and liposomes. Indeed, these were extensively investigated over the last decade regarding their potential as drug-delivery systems, due to their upgraded biocompatibility, biodegradability, low-immunogenicity, and long-term blood circulation. Finally, we conclude this work with the opened questions to address before considering exosome-like disposals for confident therapeutic delivery.

## 2. Clinical Trials Based on Exosomes

The effects of exosomes on targeted pathologies have been reviewed elsewhere [70]. Therefore, this topic will not be detailed here. However, these studies led to several clinical trials that are interesting to summarize. The first reported trials started in the year 2000, but this work only focuses on the ongoing assays (Table 1).

Technological advances, based on exosomes and applied in the field of diagnosis and prognosis, focused principally on cancer pathologies, but their potential translation and clinical settings remain under scope. Few overviews were published on this topic [71,72,73] and some clinical trials linked to this topic can be found in Table 1.

ClinicalTrials is a governmental online site (https://www.clinicaltrials.gov/—accessed on 18 February 2024) listing authorized therapeutic trials. Searching for the keyword “exosomes”, a list of 361 studies was obtained, beginning in 1999. These clinical studies are either observational, establishing the diagnostic and therapeutic character of exosomes used on human samples (serum, biopsy, etc.), or interventional (phase 2 or 3), monitoring their effects along the treatment in humans.

WHO is another website (https://www.who.int/data/stories/world-health-statistics-2023—accessed on 18 February 2024) giving access to worldwide statistics for health. Concerning the year 2023, it reports that “between 2000 and 2019, the number of deaths caused by non-communicable diseases increased by more than a third, from 31 million lives lost to 41 million lives lost, or nearly 3 out of every 4 deaths worldwide”. Additionally, it indicates that “The four major non-communicable diseases—cardiovascular disease (17.9 million deaths), cancer (9.3 million deaths), chronic respiratory disease (4.1 million deaths) and diabetes (2.0 million deaths)—collectively killed around 33.3 million people in 2019”. The breakdown of the 361 studies found on ClinicalTrials does not mirror these statistics as 141 of the trials aim at various cancers (39%), 39 concern chronic diseases such as diabetes (6.6%), hypertension (1%), or obesity (3%), and 25 concern cardiorespiratory diseases (6.9%). The table below presents the most recent clinical trials involving exosomes. Close to 56% of the 57 trials are theragnostic studies.

## 3. Artificially Loaded Exosomes

Drugs are bioactive compounds that often present low water solubility, poor organ targeting specificity, fast metabolization (degradation), easy accumulation in healthy tissues (toxicity), and poor ability to penetrate cells. Consequently, improving their delivery requests the development of reasonable carriers. As evoked in the introduction, exosomes are circulating extracellular nanovesicles derived from biological cells with a membrane structure resembling the one of the parent cells. Hence, they demonstrate low immunogenicity and high stability in the blood circulation. Consequently, the protection of the drugs and their addressing by exosomes have been considered for years. Numerous works reported that exosomes effectively protected the loaded drug from the environment, increasing their efficiency, but also enhanced their targeting capacity, and recipient cells fusing ability. It has also been noticed that, thanks to their unique membrane structure and size, exosomes easily pass through the biological barrier [74] and can escape some immunity responses [4,48]. However, introducing ingredients into pre-existing and stable structures is not simple and several strategies have been proposed.

### 3.1. Loading Methods

**Figure 2:**
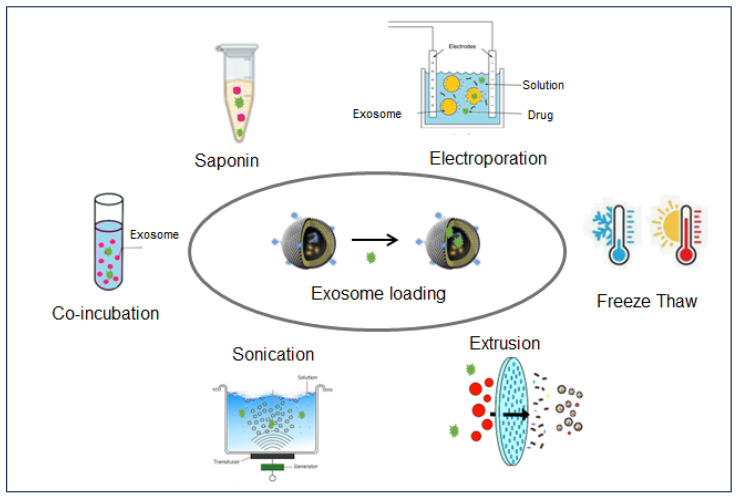
Methods for drug loading into exosomes, including co-incubation, saponin incubation, electroporation, freeze–thaw cycle, extrusion, and sonication. Different methods can be combined. The drug-loading efficiency of exosomes depends on the loading methods and conditions.

Developing an efficient and reproducible method to embed the desired therapeutic ingredient into exosomes without disturbing their biological integrity and identity is a major concern to achieve an exosome-based drug-delivery system. Two types of loading are under the scope of this paper: (1) biologically-engineered exosome production, where the ingredient is encoded by the cell that produces the exosomes’ natural cargo; and (2) the physicochemical post-loading of exosomes [75]. In this work, we reviewed only the second loading type (Figure 2).

The most employed method for small hydrophobic molecules and the easiest is the co-incubation of the drug and the exosomes at room temperature. However, co-incubation is time-consuming, has very low loading ratios (around 1%), and is not applicable to hydrophilic compounds. 

Only a few types of drugs can be passively loaded into exosomes, implying that the most employed loading methods are based on a supplementary physical or chemical force, affecting the exosomes and therapeutic cargo integrities.

Sonication and electroporation constitute alternatives to co-incubation. The sonication principle is based on the weakening of the exosome-membrane integrity by exposure to electromagnetic waves, facilitating the drug entrance. This method is compatible with hydrophilic drugs and allows a close to 30% loading ratio. The drawbacks of sonication are exosome aggregation processes and membrane disruption, both affecting the immune tolerance of the generated exosomes. 

Electroporation is a rapid and high-loading yield (up to 60%) technique. This time, electric pulses are transitory, creating micropores in the lipid bi-membrane, allowing the entrance of the chemicals. The disadvantages are significant losses of membrane integrity and possible protein destruction.

Another efficient, but less tested, method is extrusion where the drug and the exosomes have to pass together through a 100 to 400 nm membrane. During this process, the exosome membrane is destroyed and reformed, including the proximal drug molecules. All these methods are detailed and recently reviewed elsewhere [76].

The protocols that were developed are summarized in Table 2. It should be noted that the loading efficiencies reported are not fully robust. Even if authors make their best efforts to measure the drug content, the outcomes are highly variable, mostly due to the difficulty of quantifying the exosomes and of assessing if the drug is encapsulated or absorbed.

### 3.2. Loaded Exosomes Preclinical Applications

The diversity of the performed studies reflects the diversity of the ingredients that are encapsulated within the exosomes. Therefore, we choose to describe the published works in terms of the large families of compounds they transport.

#### 3.2.1. Loading for Tracking and Labelling Purposes

The marking and tracking of exosomes are necessary for setting up therapeutic applications. Optical imaging is currently the most used imaging method for tracking molecules and cells, and fluorescence microscopy is used for studying the physiological activities of living cells. For such purposes, the exosomes need to be labeled with fluorescent probes, and the physiological changes in individual cells can be monitored. Several methods of fluorescent labeling for exosomes were described. First, lipophilic organic dyes can insert the lipid exosome bilayer [77,78,79,80]. Alternatively, green fluorescent proteins can be genetically encoded in the parent cell [81]. Another approach, high-specific immunofluorescence labeling, consists of labelling the antibody with an organic dye before the exosome interaction [82]. 

Finally, exosome mimes based on trackable solid core nanoparticles with a cell-membrane shell were developed (see the paragraph exosome mimes).

#### 3.2.2. RNA-Loaded EVs

Exosomes naturally carry nucleic acids, such as DNA and RNA, to target cells in both biological and pathogenic processes [8]. Therefore, several studies were conducted to deliver genetic materials to correct diseases altering gene expression and improving genetic therapy. In particular, the successful delivery of exogenous genetic material by exosomes has been highlighted and their post-delivery functionality was described as a valuable asset [83]. The delivery of siRNA to T-cells and monocytes using human exosomes was investigated in gene therapy purposes [84]. The use of endothelial exosomes to deliver siRNA was also under scope [85]. Using electroporation, exosomes isolated from endothelial cells were loaded with siRNA designed to silence a vector encoding for luciferase (pGL2). Their efficiency in delivering siRNAs was tested on luciferase-expressing endothelial cells. The significant lowering of luciferase expression indicated that endothelial exosomes can deliver exogenous functional agents to cells in vitro. 

Another in vitro study investigated the exosome-delivery of siRNA against RAD51 (a eukaryote gene protein that assists in the repair of DNA double-strand breaks), in cancer cell lines. Exosomes were isolated from HeLa cells by ultracentrifugation, and chemically loaded with Alexa-fluor 488-labeled siRNA and then co-cultured with recipient cells (HeLa and HT1080 cells). The successful delivery efficiency of siRNA by exosomes was confirmed by confocal microscopy and flow cytometry. Western blot analysis showed a considerable reduction in RAD51 and RAD52 protein levels, indicating successful down-regulation of this specific gene [86].

Surface proteins, such as PDL-1 or CTLA-4 and FGL-1, expressed by immune and tumor cells, ensure the modulation of the immune microenvironment. Immune checkpoint therapy encounters significant clinical challenges, including low response rate to cancer treatment, acquired resistance, and immune-related adverse events. Inhibiting FGL1 and the immunosuppressive cytokine TGF-β1 is essential for colorectal cancer immunotherapy. Therefore, the authors constructed a modified erythrocyte exosome (targeting the cancer cells) and loaded them with siFGL1 and siTGF-β1. They reported a significant anti-tumor effect, both in vitro and in vivo [87]. 

The mutant form of the GTPase KRAS is a crucial driver of pancreatic cancer. The authors prepared exosomes derived from normal fibroblast-like mesenchymal cells. They demonstrated a better persistence of exosomes in the mice circulation compared to liposomes, probably thanks to a CD47-mediated protection from phagocytosis. Moreover, they loaded them with siRNA or short hairpin RNA specific to oncogenic Kras^G12D^ and reported an increased overall survival in mouse models of pancreatic cancer [88].

EVs’ delivery of siRNA was also under the scope for spinal cord injury treatments. Bone marrow MSC-derived exosomes were electroporated to load them with siRNA silencing CTGF gene. The loaded bone marrow-MSC-derived exosomes were injected into the tail vein of spinal cord injured rats. These inhibited the expressions of CTGF gene but also produced several positive side effects like quenching inflammation, decreased neuronal apoptosis, and reactive scar formation [89]. 

The peptide CAQK was chemically anchored to the membranes of EVs isolated from induced neural stem cells, after which CCL2-siRNA was loaded through electroporation. These drug-delivery systems specifically delivered siRNA to the injured region and were taken up by target cells. A synergistic effect was noticed between the inherent functions of the stem cell-derived vesicles and the loaded siRNA. Indeed, these loaded and modified EVs limited the negative effects of the inflammatory response and neuronal injury [90].

For all these studies, siRNAs were not optimized for their nanovesicular delivery, except for one work where cholesterol-conjugated siRNAs (hydrophobic siRNAs) were evaluated concerning the conservation of the activity and the loading rate onto EVs. The commercial linker triethyl glycol was preferred due to the better loading of the conjugate within the nanovesicles. It is noticed that these siRNAs-cholesterol-conjugates presented a saturation level of around 3,000 siRNA copies per EV [91]. 

In addition to siRNA, the delivery of microRNAs (short non-coding RNAs, involved in post-transcriptional regulation of gene expression in multicellular organisms by affecting both the stability and translation of mRNA) was also investigated, as exosomes are known to carry and deliver miRNA to targeted cells naturally (recent, e.g., [92]). 

Epidermal growth factor (EGF) and EGF receptor-specific peptide (GE11), which binds specifically to EGF receptor, were incorporated onto the surfaces of exosomes that carried miRNA lethal-7 gene (let-7a), to deliver let-7a to EGF receptor-expressing cancer tissue. The ability of GE11- or EGF-positive exosomes to bind to EGF receptor in various breast cancer cell lines, including HCC70, HCC1954, and MCF-7, was tested. miRNA let-7a was introduced into GE11 exosomes by lipofection. The GE11 EVs were labeled with cell-membrane PKH67 dye to track them within the recipient cells. The GE11 exosomes containing let-7a were intravenously injected into tumor-bearing mice, where they strongly inhibited the expression of HMGA2, indicating that exosomes successfully deliver their cargo to the target cells [93].

The vascular dysfunction of ovarian cancer contributes to chemotherapeutic resistance, and miR-484 was found to be down-regulated in both the cancer cells and the angiogenic endothelial cells. To enhance their tumor targeting, HEK293T cell-derived exosomes were modified to present the adhesion peptide RGD on their surface. In vivo, the injection of RGD-modified exosomes loaded with miR-484 induced vessel normalization and, in turn, sensitized the cancer cells to chemotherapy-induced apoptosis [94].

Similarly to siRNA, hydrophobic miRNA-conjugates were developed. Considering the low loading of rates miRNA within the nanovesicles, instead of engineering cholesterol-conjugates, it was proposed to conjugate miRNA with a cell-penetrating peptide. The YARA peptide, derived from human immunodeficiency virus, was covalently conjugated with miR-21-5p, and then incubated with mesenchymal stem or cancer cell-derived exosomes. The YARA-miR-21-5p demonstrated a significantly increased loading capacity and was rapidly delivered into the targeted fibroblast cells. Indeed, the loaded exosomes significantly enhanced the proliferation, migration, and invasion of human and mouse fibroblasts, which are vital steps in wound healing [95].

Another approach was to investigate the use of mi-RNA with drug co-delivery.

Triple-negative breast cancer corresponds to tumoral cells that lack human epidermal growth factor receptor 2, estrogen receptor, and progesterone receptor expressions. Therefore, only chemotherapy with platinum and untargeted chemotherapy are possible with inherent side effects. Increased binding to integrin α_v_β_3_ was obtained through a continuous protein kinase C activation in monocyte-derived macrophages, allowing obtainment of exosomes with a high metalloprotein A15 presentation. These were co-loaded with Dox and the cholesterol-miR159-conjugate to induce synergistic therapeutic effects in MDA-MB-231 cells. In vivo, this nanovesicular miR159 and Dox delivery effectively silenced the TCF-7 gene without adverse effects [96].

Similar observations/conclusions were reported regarding the anticancer 5-fluorouracil when loaded with miR-21i into 5-FU-resistant colorectal cancer cell line HCT-1165FR-derived EVs, using electroporation. Systematic administration of the so obtained exosomes in tumor-bearing mice demonstrated an anti-tumor effect. Moreover, the combinational delivery of miR-21i and 5-FU with the engineered exosomes reversed drug resistance and significantly enhanced the death of 5-FU-resistant colon cancer cells, compared with the treatment with only miR-21i or 5-FU [97].

Finally, one recent publication reports the vectorization for glioblastoma treatment using the antisense miRNA oligonucleotides loaded into modified exosomes. To enhance glioblastoma targeting, the T7 peptide binding the transferrin receptor overexpressed on the surface of glioblastoma cells was used to decorate the exosomes membrane. The antisense against miR-21 (AMO-21) was loaded by electroporation. In vitro studies demonstrated an increased AMO-21 delivery to C6 glioblastoma cells and in vivo the induction of the expression of PDCD4 and PTEN in tumors, resulting in tumor size reduction [98].

#### 3.2.3. Protein-Loaded EVs

As described for miRNAs or siRNAs, the nanoEVs can be loaded with proteins using the same methods (electroporation, freeze-thaw cycles, and/or sonication). The vectorization of several proteins, amino acids, peptides, or glycoproteins of therapeutic interest was investigated using modified nanoEVs.

Tripeptidyl-peptidase 1, also known as lysosomal pepstatin-insensitive protease (TTPI), was loaded into IC21 cell line (mouse peritoneal macrophage)-derived exosomes, using sonication. The EVs significantly increase the stability of TPPI against protease degradation and provide in vitro efficient TPP1 delivery to targeted neuronal ceroid lipofuscinose 2 cells. The majority of EV-TPP1 (≈70%) is delivered to target organelles and lysosomes. [99]. 

Nanovesicles were prepared from induced pluripotent stem cell-derived endothelial cells through an extrusion approach to function as exosome mimetics. Abundant membrane C-X-C motif chemokine receptor 4 allowed endothelial cell-targeting. Moreover, endothelial homology facilitated their accumulation in the cells. Dapagliflozin, promoting neovascularization in diabetic mice, was loaded into these bio-sourced artificial vesicles. The ability of induced pluripotent stem cells-endothelial cell nanovesicles to facilitate angiogenesis was conserved. Moreover, diabetic wound healing stimulated by HIF-1α/VEGFA pathway was demonstrated [100].

Interestingly, to increase their delivery on the injured zone, EVs were embedded into implantable biomaterials to investigate their potential for enzyme prodrug therapy. EVs derived from human mesenchymal stem cells were used as smart carriers for stabilizing enzymes in a hydrogel for a local conversion to active anti-inflammatory compounds. β-glucuronidase was co-incubated with MSC-derives EVs and incorporated into poly(vinyl alcohol) hydrogels to achieve site-specific release of curcumin (Cur) from its glucuronide precursor. The Cur formation was monitored using density-dependent color scanning electron microscopy imaging [101].

The efficiency of exosomes to deliver human insulin was studied on three different cell lines: hepatocarcinoma- (HepG2), dermal fibroblast- (HDFa), and pancreatic β (RIN-m)-cells. These cells were selected due to their glucose level regulation capacity. An autologous approach was developed, where the EVs derived from each cell line were loaded with human insulin by electroporation and internalized in cultures of their parent cells. The insulin internalization was monitored thanks to a fluorescein isothiocyanate labeling, and their glucose regulation effect was monitored by quantification of the glucose in the extracellular medium. Curiously and in contrast to HepG2 and HDFa, RIN-m cells were found to be insensitive to the loaded exosomes [102]. 

Electroporation was used to pack nanoEVs derived from donor platelets with a recombinant yes-associated protein 1. Yap1 is known to promote the stemness and differentiation potentials of tendon progenitor cells in vitro. These progenitor cells were exposed to the Yap1-loaded exosomes and differentiated toward tendon-like cells. They conserved their functions after long-term passage. The Yap1-exosomes were applied to progenitor tendon cells on a hydrogel to assist new tendon-like tissue formation in full-cut Achilles tendon defect [103].

A combination of freeze–thaw cycles and sonication was used to load human chorionic gonadotropin into exosomes isolated from uterine fluid. This hormone is a glycoprotein described to improve endometrial receptivity to implantation. The drug-release pattern and uptake of exosomes into the endometrial cells were evaluated and revealed that these loaded nanovesicles were functional [104].

#### 3.2.4. Drug-Loaded EVs

The majority of recent publications in the loaded-exosome field are focused on the delivery of chemical drugs for several pathologies, but mostly aimed at cancer therapy. 

One of precursor publications concerns a cell transfection with iRGD plasmid. iRGD is a 9-amino acid cyclic peptide (CRGDKGPDC) able to home onto tumor tissues and to bind and release doxorubicin (Dox). Exosomes were obtained from mouse immature dendritic cells that have been engineered by fusing the exosome membrane protein (Lamp2b) to αv integrin-specific iRGD peptide. Using electroporation, they were loaded with Dox. The last showed an efficient targeting and Dox delivery to αv integrin-positive breast cancer cells in vitro. When intravenously injected, these exosomes delivered Dox specifically to tumor tissues in BALB/c nude mice leading to the inhibition of tumor growth [68].

Later, Li et al. loaded one side Dox into exosomes isolated from A33-positive LIM1215 cells, and the other side coated the surface of superparamagnetic iron oxide nanoparticles with A33 antibodies. The interaction of the two produces a complex that demonstrated in vivo an excellent tumor targeting ability and the inhibition of the colon tumor growth [105]. 

Classically-activated M1 macrophages are known to suppress tumor growth. To enhance their tumor-suppressive effect, M1-exosomes were isolated from M1-macrophages and loaded with paclitaxel (Ptx), a taxoid chemotherapeutic drug, by a slight sonication. Mice bearing 4T1 breast-tumors were used to perform the therapeutic effect of Ptx-M1-exosomes in vivo, and their cytotoxicity was studied. The M1-exosomes provided a pro-inflammatory environment and exhibited an anti-tumor effect in vivo. When loaded with Ptx, the anti-tumor effects were increased [106].

Exploiting the capacity of exosomes to easily penetrate the blood–brain barrier, Ptx-loaded embryonic stem cell-derived exosomes were prepared to treat glioblastoma. The anti-glioblastoma effect of these embryonic stem cell exosomes was demonstrated. Then, exosomes were engineered by introduction of c(RGDyK) and loaded with Ptx. Their efficiency to deliver drug in glioblastoma mode was compared to the drug alone or the drug-loaded exosomes. The loaded engineered exosomes were revealed to be the best vehicles to target the glioblastoma cells [107].

To enhance pancreatic cancer therapy, surface-modified EVs were loaded with Ptx. For this purpose, human pancreatic cancer cell-derived EVs were collected. The functional ligand RGD, binding specifically to αvβ3 integrins highly expressed in pancreatic cancer cells, was conjugated to the EV surface. Ptx-loaded nanovesicles were intravenously injected into xenograft mice and produced a significant reduction in tumor size compared to the free Ptx. Interestingly, comparing the effects of RGD-modified and Ptx-loaded EVs derived from colon adenocarcinoma HT29 cells with those derived from pancreatic cancer cells, it was demonstrated that autologous EVs present more benefits [108].

Exosomes derived from human umbilical cord-derived mesenchymal stem cells promote the migration of neural stem cells NSCs in vitro and in vivo. Therefore, a collagen-scaffold was designed, including these nanovesicles to attract NSCs to enhance the therapy after complete spinal cord injury. A dual bio-specificity peptide allowed the effective retention of the exosomes within scaffolds. To complete the neural regeneration and reduce scar deposition, chemotherapy was proposed by loading these exosomes with Ptx during the extrusion process [109].

Gemcitabine (GEM), a nucleotide analog, is the first-line chemotherapeutic drug for pancreatic cancer treatment. Unfortunately, it is associated with important side effects when administered systemically. Therefore, it was proposed to encapsulate GEM within exosomes. The authors demonstrated that for pancreatic cancer, autologous exosomes (cancer cell-derived) facilitate the tumoral uptake of GEM and significantly increase its cytotoxic effect [110].

Immunotherapy in treating pancreatic ductal adenocarcinoma (like chemotherapy) does not provide satisfactory outcomes for patients due to the unique tumor microenvironment and low cancer immunogenicity. The retained approach was to reverse the tumor immunosuppression of M2-like tumor-associated macrophages through disruption of the dectin 1/galectin-9 axis. Bone marrow mesenchymal stem cell-derived exosomes were loaded with galectin-9 siRNA, by electroporation, and surface modified with oxaliplatin to assess whether they can act as an immunogenic cell death trigger. The authors found that these elicit tumor-suppressive macrophage polarization, cytotoxic T-lymphocyte recruitment, and Tregs down-regulation [111].

Ferroptosis is a form of cell death recently reported during exposure to erastin, characterized by an alteration of the cellular redox metabolism, resulting in massive lipid peroxidation in the plasma membrane. Exosomes obtained from HEK293T cells—transfected with CD47-overexpressing plasmid—were isolated and loaded with erastin and Bengal Red via sonication. Hepa1-6 cell xenograft C57BL/6 mice were injected with these engineered exosomes and the photodynamic therapy was conducted by a 532 nm laser irradiation. The CD47 surface functionalization allowed the exosomes to escape phagocytosis. In addition, the embedded erastin induced strong ferroptosis in vitro and in vivo in hepatocarcinoma cells after irradiation [112].

Chronic liver injury and liver failure caused by acute liver injury remains challenging to treat. The potential of quercetin (a flavonoid) and vitamin A loaded into adipose mesenchymal stem cell-derived exosomes was investigated to improve this fate. It was reported that vitamin A enhances the liver targeting of exosomes in acute liver injury in mice (induced by CCl4). The quercetin and vitamin A loaded mesenchymal stem cell exosomes reduced the rapid senescence-like response induced by acute liver injury [113].

Due to their high production capacity and biocompatibility, milk EVs have recently been considered to improve oral drug bioavailability [114,115,116].

The delivery to breast tissue and the anticancer activity in cell lines of free Cur and resveratrol (Rsv) were compared with encapsulated Cur and Res in milk-derived exosomes. The experiments were carried out on MCF-7 and MDA-MB-231 breast cancer and also on MCF-10A non-tumorigenic cells. The loaded milk-exosomes entered the cells primarily via clathrin-mediated endocytosis, avoiding ATP-binding cassette transporters (ABC). The milk exosomes were capable of protecting Cur and Rsv from metabolism and to deliver the polyphenols to the mammary [117]. 

The ability of exosomes to cross the blood–brain barrier and target the recipient cell was taken in account to develop a theragnostic strategy for glioma. Superparamagnetic iron oxide nanoparticles and Cur were placed into exosomes to confer imaging and therapeutic functions. After loading, the exosome membranes were chemically modified with neuropilin-1-targeted peptide (RGERPPR, RGE) to target glioma. When administered to glioma cells and orthotopic glioma models, these provided good results for imaging and therapy of glioma through magnetic flow hyperthermia and Cur chemotherapy [118].

However, many drugs have poor oral bioavailability, warranting the use of injections. The capacity of cow milk EVs and intestinal epithelial cell-derived EVs to deliver oral drugs was studied. EVs, fluorescently labelled and loaded with Cur were tested in an intestinal model (Caco-2). Epithelial cell-derived EVs showed notably higher cell uptake compared to cow milk EVs. However, the cell uptake was significantly higher in differentiated compared to undifferentiated cells for both types of EVs [119].

Finally, a singular work reported on the vectorization of antibiotics using nanoEVs. *Staphylococcus aureus* survivals inside phagocytes constitute bacterial reservoirs relatively protected from antibiotic treatments. Linezolid was loaded into exosomes produced by mouse RAW264.7 macrophages to overcome intracellular infections by pathogenic bacteria. This exosomal formulation of linezolid was effective in vitro and in vivo without evident cytotoxicity in macrophages [120].

## 4. Artificial Mimes for Exosomes

Despite the large number of studies carried out using exosomes isolated from cell cultures, one cannot ignore the major issues linked to their production and purification.

One of the major problems encountered for exosome-based drug-delivery systems is the low productivity of exosomes. Indeed, the typical reported production yields are maximally of 1 μg exosomal protein/mL of culture medium [121], whereas the typical therapeutical dose used was of 10–100 μg exosomal protein/mouse [122]. EVs are usually prepared by culturing the parent cells and seem to reach their upper production-limit after around 12 h of incubation (time depending on the parent cells) [123]. Bioreactors are reported to improve the yield of exosomes [124,125,126]. However, this quantitative improvement comes at the expense of quality, since larger vesicles are obtained. Another way to enhance the production yield is to apply stresses (hypoxia, low pH, drugs, …) on the parent cells. Unfortunately, it was demonstrated that cellular stresses changed the composition of exosome cargo and surface [127]. Moreover, these increased contaminants such as apoptotic bodies. 

Conventionally, exosomes are isolated through ultracentrifugation-based methods, but due to their size and density, these are contaminated with proteins and are often fusing or aggregating under extreme physical constraints [121]. Therefore, other exosome-isolation methods have been explored, such as size exclusion chromatography (SEC), ultrafiltration (UF), or polymer-based precipitation (PP). More adapted for the large-scale production of exosomes, these also have disadvantages (UF—low purity, damages EVs by shear stress; SEC—low capacity, high dilution of samples; PP—low purity, severe EV aggregation) [128,129,130]. The recent technical evolution involves microfluidics [131].

Another issue comes from the observation of subpopulations within the nanoEVs, which differed in their composition and size [132]. Different compositions of proteins and RNAs in exosomes, dependent on the collection method, were reported [133,134]. This can be explained by enrichments in various subpopulations, contaminants or by the presence of the protein corona that is adsorbed on the nanoparticle surface along the purification/isolation procedure. One has to note that it is unclear whether the observed protein coronas are natively present on exosomes or are the result of the isolation procedure. In any case, they clearly change the properties of nanoparticles [135,136]. Therefore, optimization of the isolation method is important to preserve the properties of exosomes and to reduce the risk of side effects induced by contaminants or specific subpopulations.

Alternative solutions have been proposed considering the (hemi)synthetical production of exosome-like structures (ex vivo).

**Figure 3:**
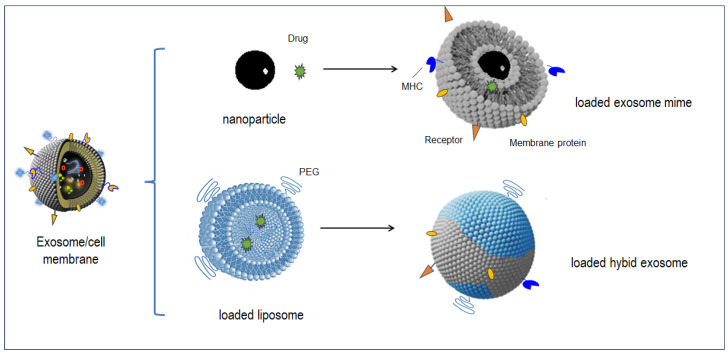
Exosome mimes are constituted by cell or EV membrane elements that are covering nanoparticles or that are mixed with liposome ingredients. These strategies allow to take advantage of 1) the shape and tracking-ability of the nanoparticles, or 2) of the loading capacity of liposomes, and 3) retains the exosome capacity to escape immune reactions and to target the recipient cells for drug delivery.

Lipid-based nanovesicles are difficult to obtain through spontaneous organization, especially when membrane proteins must be integrated. As the presence of membrane proteins is necessary for cellular targeting and internalization, innovative strategies were elaborated to include recognition elements on the surface of synthetic nanoparticles (Figure 3).

The most exploited liposome-alternative approach consists of the so-called cellular nanoparticles (cNPs). These are cell-membrane coated nanoparticles produced by a series of sonication and extrusion of cell membrane debris and vesicles. Indeed, this approach allows the cNPs to contain the lipids and proteins of the source cells and consequently exhibit several properties of the exosomes and parent cells. These have a potential variety of biomedical applications, including drug delivery, phototherapy, combination therapy, immune modulation, sensing, and diagnosis [137,138,139]. For example, it was reported that platelet-derived cNPs still interact with circulating tumor cells and bind to the vessels and tissues [140,141,142]. It was also observed that cNPs obtained from M1 macrophage membranes can repolarize tumor activated macrophages to an M1-like phenotype [106,143]. 

Interestingly, based on exosome’s spherical structures, the designed mimics often have the same shape. Convinced that rod structures are more appropriate to penetrate tissues and to be endocytosed, Zhang et al. developed cancer cell membrane-coated nanoparticles with the two shapes. Thanks to the caveolin-mediated pathway, the coated nanorods were more efficient for endocytosis and in the endoplasmic reticulum region accumulation compared to the spherical ones [144].

### 4.1. Cell-Membrane Covered Napoparticles

#### 4.1.1. Single-Membrane Covering

Artificially produced bio-sourced nanoparticles have received growing attention in drug delivery. Because of anti-cancer drugs’ toxicity, the tumor-specific delivery enabled by the cNPs constitutes the most part of the published exosome-mime applications, as they allow the delivery of the drugs of interest at much lower concentrations to target sites, with limited side effects (see also loading part) (Figure 3). 

As erythrocytes represent the most abundant blood circulating cells, these are the first biological source of reported cellular NP-coatings but are still under scope to ensure a ready clinical translation. Indeed, a higher membrane extraction rate must be obtained for a potential scaling-up, and the encapsulation efficiencies should be improved to provide batch-to-batch reproducibility [145]. Early in 2013, polymeric nanoparticles were masked using erythrocyte membranes [146] and were used to deliver drugs, especially Dox and Ptx [147,148,149]. Among them, the most surprising approach was the direct encapsulation of Ptx spherical crystals with erythrocyte membranes. Due to the high drug content, the authors achieved a stronger cytotoxicity and higher drug accumulation in tumor. Therefore, they claimed that it is the most effective Ptx formulation against tumor growth among all in tumor-bearing mice models [149].

**Table 2:** Drug-loading methods for nanoEVs found in the literature.

Loaded Molecules	Exosome Cell Line	Loading Methods	Max Loading Efficiency/Capacity	Targeted Pathology/Fonction	Ref.
siRNA MAPK1	HeLa, HTB 177, human blood cells	Electroporation: 150 V-100 µF	NS	Cancer therapy	[84]
siRNA luciferase	Primary endothelial cell mice	Electroporation: 400 V-200 µF	25%	Drug absorption	[85]
siRNA RAD51 and RAD52	HeLa, ascites	Electroporation: 700 V-350 µF	NS	Drug absorption	[86]
siRNA FGL siRNA TGF-β1	RAW 264.7	Transfection with commercial reagent	97%	Cancer immunotherapy	[87]
siRNA KRASG12D	Human MSC	Electroporation: 400 V-150 µF	NS	Pancreatic cancer	[88]
siRNA CTGF	Rat MSC	Electroporation: 400 V-200 to 500 µF	33%	Cancer therapy	[89]
siRNA CCL2	Induced neural SC, surface modified with CAQK	Electroporation: 400 mV-125 μF (pulse time 10–15 ms)	17%	Spinal cord injury	[90]
siRNA DNSL1; SND1; ENDD1; RINI; 5NTD; ENPP1; CN37	Umbilical cord Wharton’s jelly mesenchymal stem cells	Co-incubation, 37 °C, 1 h	18%	Huntington’s disease	[93]
miRNA 484	HEK293T	Electroporation: 700 V-150 µF	NS	Metastatic ovarian cancer	[94]
miRNA 21-5p (conjugated to CPP)	Mouse embryonic fibroblasts (MF) Human primar dermal fibroblasts (HDFa)	Co-incubation, 37 °C, 8 h	1600 copies/exo	Loading optimization	[95]
miRNA 159 doxorubicine	Human monocytes THP-1	Co-incubation, 37 °C, 1 h 30	16%	Breast cancer	[96]
miRNA 21 inhibitor 5-FU (5-fluorouracile)	Transfected HEK293T cells	Electroporation: 1000 V-15 µF, 10 ms	3%	Colorectal caecinima	[97]
miRNA 21 antisens	Transfected HEK293T cells	Electroporation: 400 mV-125 μF	1.7%	Glioblastoma	[98]
TPP1-encoding *p*DNA	Macrophages	Sonication; saponin-permeation	NS	Late-infantile neuronal ceroid lipofuscinosis	[99]
Dapagliflozin	iPS-derived ECs	Extrusion	28%	Diabetic wound healing	[100]
β-glucuronidase	Human MSCs	Saponin permeation: 0.1 mg/mL, 10 min, RT	NS	EV-based hydrogel-developments	[101]
Human insulin	Hepatocellular carcinoma (HepG2) Primary dermal fibroblasts (HDFa)	Co-incubation, RT, 1 h Electroporation: 200 V-50 µF	3% 50%	Diabetes melitus treatment	[102]
Yap1 protein	Rat plasma platelets	Electroporation: 200 V-500 µF, 26 ms	NS	Achilles tendon injury	[103]
Human chorionic gonadotrophin	Human uterine fluid	Freeze-thaw cycle and sonication	40%	Assisted reproductive technology	[104]
Doxorubicin	Human breast cancer cells (MDA-MB-231, MCF 7)	Electroporation: 350 V-150 µF	NS	Tumor therapy	[68]
Doxorubicin	Huma CRC cells LIM1215	Incubation: 5 min Dialyse PBS overnight	9%	Colorectal cancer	[105]
Paclitaxel	M1 macrophages RAW 264.7	Sonication: 20% amplitude; 6 cycles of 30 s	NS	Cancer therapy	[106]
Paclitaxel	ESC H9 cells	Incubation: 2H; RT	NS	Glioblatoma	[107]
Paclitaxel	PANC-1 (pancreatic ductal carcinoma) cells; U937	Co-incubation, RT, 1 h and sonication	37%	Pancreatic cancer	[108]
Paclitaxel	Human umbilical cord-derived mesenchymal stem cells	Extrusion	14%	Spinal cord injury	[109]
Gemcitabine	Pancreatic cancer cells	Incubation: 2H; 37 °C Sonication: 20% amplitude; 3 cycles of 30 s	12%	Pancreatic cancer	[111]
siRNA galectin-9 oxaliplatin	BM MSC cells	Electroporation: 400 V-125 µF	13%	Pancreatic cancer	[112]
Erastin rose bengal	CD47-overexpressing HEK293T	Sonication	60% 84%	Hepatocarcinoma	[113]
Vit A + quercetin	Mice MSC	Incubation: 2 h; 22 °C	Vit A: 0.279 µg/mL exo Que: 0.19 µg/mL exo	Acute liver injury	[114]
Curcuma resveratrol	Raw bovine milk	Incubation: 1 h, 37 °C darkness Sonication: 1 Hz; 150 W; 4 or 6 cycles of 20 s. Electroporation: 400 V; 2 pulses	Cur 2 µg/0.5 ng prot exo; Res 15 µg/0.8 ng prot exo	Breast cancer	[119]
Curcuma	Cow skimmed milk Intestinal cells (Caco-2)	Incubation: overnight; RT Size exclusion chromatography	Cur:9% Caco-2: 3%	Drug absorption	[149]

NS: not specified, RT: room temperature.

Since immunotherapy remains a powerful cancer therapy, the first generation of cNPs were designed using immune-cell membranes.

Employing the “cell ghosts” (nanoghosts) production protocol, core-shell nanoparticles in the 100–200 nm size-range were obtained using serial extrusions of cellular membranes through a polycarbonate membrane. The coating of monocyte cell membrane on a biodegradable poly(latic-*co*-glycolic acid) core formerly loaded with Dox was reported, and the intracellular uptake of these cNPs into metastatic breast niches was tested using MCF-7 cells. Comparing FITC-loaded cNPs with FITC-loaded PLGA NP controls, a higher cellular uptake was observed for the first ones. No observable toxicity towards MCF-7 free cells could be seen [150]. 

The same core-shell disposal was reported, exploiting this time the property of inflammatory neutrophils to target the circulating tumoral cells and the metastatic niche. The neutrophils membrane associated with the poly(latic-*co*-glycolic acid) nanoparticles demonstrated a highly preserved binding capacity to 4T1 cell models in vitro, and a high circulating cancer cells capture efficiency in vivo. Moreover, these cNPs were found to have improved homing to the premetastatic niche. When loaded with carfilzomib, they depleted the tumoral cells circulating in the blood selectively [151]. Finally, similar cNPs were reported to target hepatocarcinoma cells. The drug-free coated nanoparticles were described as highly biocompatible with a cancer-cell specificity. When loaded with Cur, the cNPs were revealed to be effective cancer-cell killers with a potent anti-tumor activity in a murine H22 model [152]. 

In addition to the cancer studies, a single report on HIV has been published. T-cell-membrane-coated nanoparticles with a polymeric core have been designed. These still present the T-cell antigens that are critical for HIV binding (CD4 receptor and CCR5 or CXCR4 coreceptors). The cNPs demonstrated a selective binding with the HIV glycoprotein gp120, inhibiting the gp120-induced killing of bystander CD4+ T-cells by a decoy strategy [153]. 

In the literature, tumor-addressing is almost ensured through a cancer cell membrane encapsulation or core coating.

A mesoporous silica nanoparticle-supported PEGylated liposome core has been developed, enabling the coating with various cancer cell membranes. The moderate rigidity of these cNPs cores facilitated their penetration throughout multicellular spheroids in vitro, indicating a probable facilitated infiltration into the tumor. They were directly internalized by membrane fusion, and the PEGylated cores were released into the cytosol. Their ability to co-encapsulate Dox and mefuparib hydrochloride has also been tested [154].

Recently, in addition to the NP masking with glioma C6 cancer cell membranes, the retroenantiomer of quorum-sensing peptide (WSW)—which can efficiently cross the blood–brain barrier—has been inserted. The so-obtained nanosuspensions were not cleared by the immune system, could cross the blood–brain barrier, and targeted the tumor selectively [155]. Another way to cross the blood–brain barrier for the cNPs was followed by exploiting the penetration ability of metastatic 4T1cancer cells. Following this approach, a biomimetic nanoplatform was designed by masking a succinobucol-loaded pH-sensitive polymeric particle with a 4T1 cell membrane to promote the preferential targeting of cerebral ischemic lesions [156].

To obtain nanoparticles capable of targeting oral squamous cancer cells, membranes of mesenchyme stem cells obtained from dental pulp were used to cover metal-organic nanoparticles. As they present the CXCR2 receptor, these particles possessed the attempted specificity for the targeted cancer cells. Moreover, when loaded with Dox, the cNPs could induce CAL27 cell death in vitro and blocked CAL27 tumor growth in vivo [157]. 

#### 4.1.2. Hybrid Membrane Covering

The production of nanovesicles derived from two different types of cells has been published, resulting in a novel cNP family called hybrid nanovesicles, that inherit the characteristics from the two source cells (Figure 3).

cNPs derived from monocytes were used to deliver Dox into SKOV-3 ovarian cancer cells. Using confocal microscopy and flow cytometry the authors proved that such mimetic disposals are more efficient than free Dox, by reducing side effects and increasing its cytotoxicity for the targeted cells. The comparison of exosome mimes with the natural exosomes produced by these cells demonstrated the conservation of the exosome markers profile (CD81, CD63) with a reproducible and 2.5 times more efficient production yield for the mimes [158]. 

Although chemotherapy is a promising strategy for bone invasion treatment, its clinical applications are limited by the lack of tumor-specific targeting and poor permeability in bone tissue. Therefore, developing a smart bone and cancer dual targeting drug-delivery platform is necessary. Spherical nanoparticles designed for bone and cancer targeting were covered with a head and neck squamous cell carcinoma WSU-HN6 cell (H) and red blood cell (RBC) hybrid membrane. These were modified by an oligopeptide of eight aspartate acid (Asp8) and exhibited the same surface proteins as those of WSU-HN6 and RBC. In vivo, these nanoparticles were mainly localized proximal to the cancer cells. Moreover, they showed effective cancer growth-inhibition properties when compared to other formulations [159].

Another idea was followed by preparing EV mimics presenting the macrophage signal regulatory protein alpha SIRPα (SαV-C-nanovesicles) to disrupt the CD47-SIRPα axis releasing a “do not eat me” signal to prevent the phagocytosis of cancer cells. As the authors aimed to repolarize TAMs towards the M1 phenotype simultaneously, they designed hybrid cell membrane nanovesicles displaying SIRPα variants and containing M2-to-M1 repolarization signals. These hybrid cell-membrane covered nanovesicles lead to potent tumor inhibition in a poorly immunogenic triple negative breast cancer model [160].

#### 4.1.3. Membrane Covering of Organometallic Cores

Besides the structural function of the solid nanoparticles allowing for a fixed size and shape of the cNPs, some solid inner cores were also designed to play the therapeutic role. 

In 2017, an early publication reported the possibility of using platelets as carriers for gold nanorods. These were loaded into platelet cells by electroporation. The resulting loaded-gold particles retain the long blood circulation and cancer-targeting characteristics of the unloaded cells. They also present the good photothermal property of the gold rods [161].

Later, the same group designed a platelet-cancer stem cell hybrid membrane-coated iron oxide magnetic nanoparticle. The platelet membrane conferred the immune evading ability, and the cancer stem cell membrane conferred the homotypic targeting capabilities, thanks to specific surface adhesion molecules. The hybrid covering of the magnetic particles enhanced the photothermal therapy of head and neck squamous cell carcinoma, inhibiting tumor growth in mice [162].

Coating upconverting particles—used for photodynamic therapy and imaging purposes—with macrophage membranes makes it possible to utilize the adhesion between macrophages and cancer cells for effective cancer targeting. Natural macrophage membranes, along with their associated membrane proteins, were reconstructed into vesicles and then coated onto synthetic upconverting NPs. The resulting macrophage membrane-camouflaged particles exhibited effective cancer-targeting capability inherited from the source cells and were further used for enhanced in vivo cancer imaging, with a good in vivo biocompatibility [163]. Indeed, the introduction Fe_2_O_3_ in iron metal nodes can effectively catalyze the Fenton reaction to produce hydroxyl radicals (^•^OH) and overcome the hypoxic environment of tumor tissue by generating oxygen, improving tumor therapy. Such organometallic framework nanoparticles were camouflaged with erythrocyte membranes to enhance blood circulation and tissue residence time in the body. Moreover, the aptamer of the targeted AS1411 molecule was added to the surface to address the photosensitizers on the tumor domain. It was reported that the camouflage by the erythrocyte membrane could reduce side effects and improve the therapeutic effect of photo-dynamic therapy and chemo-dynamic therapy [164]. 

Even if the membrane camouflage remains a consensual approach, conjugates of small anticancer drug molecules with the polyzwitterion poly(2-(*N*-oxide-*N*,*N*-diethylamino)ethyl methacrylate) were recently designed, exhibiting a long blood-circulation half-lives and producing negligible interaction of the polyzwitterion with proteins and phospholipids. Adsorption of the polyzwitterion–drug conjugates to tumor endothelial cells and then to cancer cells favored their infiltration into tumors in mice. The simplicity and potency of the polyzwitterion–drug conjugates should facilitate the design of translational anticancer nanomedicines [165].

### 4.2. Exosome–Liposome Hybrids

As evoked above, the drawbacks of developing exosomes for drug-delivery purposes are related to their low biological production, the poor yield of the current isolation techniques, their limited colloidal stability at ambient environment, and their aggregation occurring during storage. The polymeric or metallic exosome-alternative nanoparticles present better stabilities and higher loading capacity but have immunogenicity, long circulation, and toxicity issues that were partially addressed through cell membrane covering strategies. Moreover, the limitations of conventional isolation techniques, such as ultrafiltration, ultracentrifugation, precipitation, microfluidics-based separation, and immune affinity-based separation, are numerous. Indeed, aggregation phenomenon, loss of integrity of exosomes due to mechanical stress, and low purity due to entrainment or adsorption effect of proteins or small hydrophobic molecules are hazards often reported [166,167]. 

To limit these drawbacks, fused liposome–exosomes are proposed alternatives for developing drug-delivery systems. Indeed, these artificial structures bring together the biological functions, targeting, and high loading capacities of exosomes, with the improved colloidal stability, and the easy surface modification of liposomes. Owing to their similar bilayer lipidic structures, exosomes and liposomes can easily fuse into hybrid exosomes (HEs) [168]. 

Despite the fact that HEs were supposed to alter the efficiency of the interactions with the cells—considering the modification of the membrane lipids composition—several studies demonstrated their high potential for DDS (Table 3).

#### 4.2.1. Hybridization Techniques

The freeze–thaw method is a valuable reported method to fuse membranes with liposomes. Its principle is based on the temporary disruption of the plasma membrane by ice-crystal formation, allowing water-soluble molecules to penetrate the structure. A high fusion efficiency is reported. Usually, cell-derived exosomes are mixed with the liposomes in a 1:1 ratio. The mixture is then frozen in liquid nitrogen and thawed subsequently at room temperature. The duration and number of freeze–thaw cycles are variable (e.g., [169,170]). The disadvantage of this method is that the high-frequency freeze–thaw cycles affect the biological activity of pharmaceuticals and also alter the integrity of the exosome membrane.

Therefore, some authors preferred natural incubation, to preserve the vesicles and drugs, but the fusion efficiency under these conditions is relatively low and time-consuming (e.g., [171]).

The most frequently used technique remains extrusion, where exosomes and liposomes simultaneously pass through membrane pores under physical pressure to form mixed vesicles at the exit. The principal advantage resides in the size homogeneity of the hybrid vesicles. Usually, the two ingredients are vortexed and sonicated before the extrusion through 100–400 nm pore-sized polycarbonate ester films. The extrusion has to be repeated (up to 10 times) [172].

#### 4.2.2. RNA-Loaded HEs

The limited drug-loading capacity of exosomes and the unclear intracellular transport mechanism may affect the transfection efficiency of exogenous nucleic acids. Therefore, the integration of artificially synthesized exogenous lipids into isolated exosomes to deliver siRNA was expected to provide a promising therapy for genetic diseases.

Considering that the complex components of tumor cell-derived exosomes contain key components of carcinogenesis, membrane fusion hybrid exosomes were engineered by fusing membranes of EVs derived from SK-Hep1 hepatic adenocarcinoma cells with phospholipids. Cyclin-dependent kinase 1 (CDK1)-siRNA (which inhibits the CDK1 gene to kill c-Myc-overexpressing hepatocarcinoma cells) was loaded using electroporation. Hybrid exosomes achieved stable siRNA encapsulation, precise tumor targeting, and effective gene silencing by the delivered CDK1-siRNA [173].

Osteoporosis is a public health problem linked to the aging population. In this context, NIH-3T3 cells were genetically engineered to enhance their CXCR4 expression. The CXCR4+ exosomes derived from these cells were fused with liposomes carrying the miRNA-188 inhibitor to produce hybrid nanoparticles. These HEs specifically accumulated in the bone marrow and released antagomir-188, thus promoting osteogenesis and inhibiting adipogenesis in bone marrow MSC [174].

Similarly, siRNA-AF647 was added during the hydration of the liposome film. Then cardiac progenitor cell-derived exosomes were mixed with the loaded liposomes, and hybrid exosomes were obtained through extrusion. The designed hybrid exosomes reduced the toxicity of liposomes and retained the gene silencing effects. Upon fusion, the properties of cardiac progenitor cell-derived exosomes were also preserved, such as the ability to activate endothelial signaling pathways and stimulate microvascular endothelial cell migration, which may have therapeutic potential for salvaging myocardial tissue upon infarctions [175].

#### 4.2.3. Protein-Loaded HEs

The clustered, regularly interspaced short palindromic repeats (CRISPR)/CRISPR-associated protein 9 (Cas9) system has become essential for in vivo gene edition. Nevertheless, challenges persist in its clinical application. The CRISPR/Cas9 system is mainly delivered to the body through viral vectors, questioning its clinical application. The expression plasmid of Cas9 is much larger than the small nucleic acids usually packed into exosomes. The delivery of such large plasmids via hybrid exosomes was studied based on the high plasticity of liposomes. Large nucleic acids, including CRISPR/Cas9 expression vectors, could be encapsulated into hybrid exosomes derived from mesenchymal stem cells, opening new possibilities for genetic manipulations in vivo [172].

#### 4.2.4. Drug-Loaded HEs

Hybrid nanovesicles obtained by fusing liposomes and tumor-derived extracellular nanovesicles (4T1 breast cancer and B16F10 melanoma cells) were successfully developed to deliver Dox for immune chemotherapeutic purposes. This engineered drug-delivery carrier obtained by extrusion techniques produced effective immunogenic cell death induction for the tumor microenvironment. The hybrid nanovesicles loaded with Dox exhibited synergistic antitumor effects on multiple tumor models [176]. Compared to the loaded tumor-derived exosomes, the lipid-modified exosomes displayed long-term colloidal steadiness and high encapsulation efficacy without any apparent burst release of drug under normal physiological conditions [177]. 

Rayamajhi et al. were the first to fuse mouse macrophage J774A.1-derived exosomes with liposomes and to load them with water-soluble chemotherapeutic drug Dox. Taking advantage of the tumor-targeting properties of macrophage membranes, the Dox-HEs demonstrated an enhanced targeting ability and cytotoxicity to J774A.1, K7M2, 4T1, and NIH/3T3 cells, when compared to Dox-loaded liposomes [178].

Considering the exosome capacity to cross the blood–brain barrier, HEs obtained from the sonication-induced fusion of salvianolic acid B-loaded blood exosomes with cryptotanshinone (CT)-loaded liposomes were proposed to treat glioma. On the one hand, the exosomes were isolated from rat serum, and the SAB encapsulation was obtained via electroporation. On the other hand, truncated Lyp-1 peptide CTP was included during DSPE/PEG-liposome formation. The tLyp-1 peptide enabled the capture and accumulation of the HEs in vitro in human glioblastoma astrocytoma cells (U87) and in deep tumor regions in vivo on BALB/c nude mice bearing U87-Luc xenograft tumors, resulting in intensive drug accumulation at glioma sites. As these HEs transported bi-therapeutic agents (SAB and CPT), synergistic anti-glioma mechanisms with two separate compartments of glioma, cancer cells and angiogenesis, were observed [179].

Pulmonary fibrosis is a progressive and fatal lung disease, but most drugs cannot effectively hinder its progression due to their poor organ targeting. Therefore, hybrid exosomes were designed by fusing chlodronate-loaded liposomes and fibroblast-derived exosomes. The loaded clodronate depleted hepatic macrophages to reduce the hepatic uptake and utilized the homing of autologous exosomes to deliver the anti-fibrotic drug Nintedanib (Nin). The authors reported the massive accumulation and penetration of HEs in pulmonary fibrosis, enhancing the suppressive effect of Nin on fibrosis [180].

Similarly, this team also developed HEs to increase, this time, the efficacy of the chlodronate inhibition of Kupffer cells and to deliver selectively Nin to liver fibroblasts [181].

Finally, another type of cancer bi-therapy combining a chemotherapeutic drug and a miRNA was proposed using HEs. Indeed, to circumvent ovarian cancer resistance to chemotherapy during chemotherapy, SKOV3-CDDP tumor cell-derived exosomes were fused with liposomes including tumor targeting peptides. The HEs were engineered to encapsulate triptolide and miRNA-497. Tumor targeting was ensured through homologous targeting, CD24-overexpession on the exosome membrane, and by the inclusion of cRGD peptide in the liposome part of the hybrid structure. It was reported that these hybrid nanoparticles effectively targeted tumor sites. In the tumor microenvironment, miR497 and tripotolide were rapidly released, inducing synergistically the apoptosis of ovarian cancer cells and the inhibition of the PI3K/AKT/mTOR signaling pathway [182].

Photothermal therapy (PTT) is a recent but widely used cancer therapy. It can destroy tumor cells through hyperthermia but also achieve effective controlled release of drugs. Hyperthermia is generated by a laser irradiation of the tumoral accumulated photothermal agents.

It was proposed to engineer CD47-overexpressing CT26 colorectal carcinoma cells to produce CD47-enriched EV membranes capable of targeting carcinoma tumors and ensuring safe circulation in blood. In parallel, a liposome containing indocyanine green as a photothermal agent and R837 (an imidazoquinoline amine) as an immune adjuvant was produced. Finally, the engineered EVs and liposomes were fused. The so obtained HEs were described as taking advantage of the two systems: the stable blood circulation, tumoral targeting, improved the macrophage-mediated phagocytosis of tumor cells (by blocking CD47 signaling) with a photothermal destructive capacity [159].

Following the same strategy, platelet-derived exosomes were combined with photothermal-sensitive DPPC/DPPG liposomes, encapsulating this time glucose oxidase (GOx) and ferric ammonium (FA). GOx was used to oxidize glucose to produce hydrogen peroxide and FA to catalyze cascade reactions, enhancing the chemotherapy efficacy. Moreover, the photothermal effect further enhanced the therapeutic effect. This hybrid system was reported to significantly reduce tumor size and prolong lifespan in treating primary or metastatic tumors [183]. 

Unfortunately, ovarian hyperthermic chemotherapy is not efficient within the peritonea, as the chemical agents are not capable of penetrating the large tumors. To overcome this issue, BALB-3T3 fibroblasts were genetically engineered to produce CD47-rich exosomes, and thermosensitive liposomes were loaded with granulocyte-macrophage colony stimulating factor or docetaxel. The two were freeze–thaw fused. It was reported that, after intravenous administration, the HEs accumulated in tumors and released their therapeutic ingredients. The intraperitoneal chemotherapy of ovarian cancer was described as accelerated under the hyperthermic condition. These hybrid exosomes effectively inhibited the tumor progression [184].

**Table 3:** Exosome hybridization and loading methods found in the literature.

Exosome-Donor Cells	Liposomes	Cargo	Fusion Technique	Application	Ref.
RAW 264.7 macrophages CMS7 sarcoma fibroblast	DOPC or DOPS	Fluorescent DMPE	Freeze–thaw	Setting-up hybridation	[170]
HEK293 T embryon cells	Not mentioned	Crispr/Cas9	Co-incubation	Gene therapy	[172]
A549 and 3C3 cancer cells	DOPC/DOPS	Anti siRNA GFP	Membrane extrusion	Setting-up for gene delivery	[173]
K-HEP1 cancer cells	DPPC	siRNA CDK1	Membrane extrusion	Hepatocarcinoma	[174]
NIH-3T3 CD24++ cancer cells	DOTAP/cholesterol	Anti miRNA 188	Membrane extrusion	Osteoporosis	[175]
SKOv3 cancer cells CP cardiomyoblasts	DLin-MC3-MA/DPPC/cholesterol/18:1-BiotinylCapPE/DMG-PEG	siRNA AF647or AF488	Membrane extrusion	Cardiac injury	[176]
4T1 breast cancer cells B16F10 melanoma	DOPC/cholesterol/mPEG_2000_/DSPE	Doxorubicin	Sonication and extrusion	Cancer immunotherapy	[177]
J774A.1 macrophages	Egg-PC and cholesterol	Doxorubicin	Membrane extrusion		[179]
Rat blood-serum	DSPE-PEG_2000_-tLyp-1	SAB in exosome tLP1 and CTP	Membrane extrusion	Glioma	[180]
L-929 fibroblast raw 264.7 macrophages	Cholesterol/DOPC/DSPE-PEG_2000_	CLD and Nin	Membrane extrusion	Pulmonary fibrosis	[181]
L-929 fibroblast	Cholesterol/DOPC/DSPE-PEG_2000_	CLD and Nin	Membrane extrusion	Liver fibrosis	[182]
SCOV3-CDDP CD24++ cancer cells	DSPE-PEG_1000_-COOH	cRGT, tripotolide and miRNA-497	Membrane extrusion	Ovarian cancer	[183]
Mouse blood platelets	Thermosensitive liposome: DPPC/DPPG/cypate	Glucoxidase and ferric ammonium	PEG mediation	Hepatocarcinoma	[184]
3T3 CD24++ cancer cells	Thermosensitive liposome: CFL/DPPC/MSPC/DSPE-PEG_2000_	GM-CSF and docetaxel	Freeze–thaw	Metastatic peritoneal carcinoma	[50]

DOPC—dioleoylphosphatidylcholine; DOPS—dioleoylphosphatidylserine; DPPC—dipalmitoylphosphatidylcholine; DOTAP—1,2-dioleoyl-3-trimethylammonium propane; DLin-MC3-MA—(6Z,9Z,28Z,31Z)-heptatriaconta-6,9,28,31-tetraen-19-yl 4-(dimethylamino)butanoate; DMG—dimethylglycine; PEG—polyethylenglycol; DSPE—disterarylphosphatidylethanolamine; DPPG—dipalmitoylphosphoglycerol; MSPC—myristoylstearoylphosphatidylcholine; CFL—cerasome forming lipid.

## 5. Perspectives

Since exosomes were identified quite early as high-potential drug-delivery vehicles, this review primarily focuses on recent nanovesicular loading strategies for nucleic acid, protein drugs, and small molecules. However, the last decade demonstrated that exosome-mediated drug-delivery methods suffer from low mass production, delivery rates, and high cost. One supplementary important issue remains that the specific functions of exosomes are not fully understood, making it challenging to ensure their long-term safety and efficacy. Moreover, one must remember that exosomes have different membrane and cargo compositions and biological functions, depending on source cells and environmental conditions, inducing reproducibility issues. For all these reasons, they cannot be considered as simply acting like empty carriers, especially when they are derived from circulating cancer or infected cells.

Therefore, the potential of exosome-mimes or analogues was studied more recently. Nevertheless, considering exosomes or exosome-like vesicles, the loading of exogen therapeutic agents is almost necessary. Unfortunately, gentle co-incubation is inefficient, and the alternative techniques partly damage the vesicles. Therefore, it is essential to ensure consistency of exosomes and drugs structure and function.

We especially focused on the recent developments of exosomes-mimes, because they open new lines considering the important issues that are required to be addressed before exosomes can be approved for clinical use. Although exosome-based therapeutics have succeeded in numerous studies, several hurdles also remain. We will announce them hereafter, to put into perspective the path that remains to be taken before their clinical application

Enhancing EV secretion is one of the challenges, and several approaches have been developed, such as culturing under turbulent fluids, under acidic conditions, under salty stress, etc. However, it is important to note that exosome composition is dependent on the environmental conditions. This questions the conservation of the attempted function under these near-to-lethal stresses.

Even if exosomes have intrinsic addressing capacity thanks to surface components and autologous recognition, important research energy was dedicated to further modifying their surface with supplementary targeting ability or extended circulating half-life. Such approaches are developed for EVs, but also for the exosome-mimicking particles. This time, it must be noticed that such approaches can potentially induce immune reactions, especially if these peptides can be systemically released. Consequently, enhancing the anchoring of such targeting peptides on exosomes is a real issue. 

Another line of work would be to develop the embedding of exosomes and exosome-like structures in biocompatible hydrogels to control their release, extend their residence time at the applied site, and optimize cell targeting through proximity.

The synthetic exosomes that can mimic the characteristics of natural exosomes are under scope but have not been as extensively investigated as modified exosomes. Two promising approaches are yet to be developed: (i) the cell or exosome membrane coating of organic or metallic particles; and (ii) hybrid exosomes, taking the advantages from the exosomes and liposomes. These engineered exosomes can carry different therapeutic agents and present specific targeting fragments on their surface, providing possible pluripotent vectorization platforms. However, concerning the HEs, controlling the fusion of liposomes and cell debris, as well as preventing the fusion between liposomes or hybrid exosomes, remain important issues that need to be addressed. 

Moreover, if hybrid structures are not easily cleared by the mononuclear phagocytic or reticuloendothelial systems, this allows the improvement of drug-delivery efficiency, bringing into question their long-term accumulation and linked toxicity. To our knowledge, no work has been published in this direction.

Finally, one major issue for the clinical validation of exosomes or exosome-like structures resides in the purity of these (loaded) carriers. The surface adsorption of medium proteins, modification agents, or drugs is almost kept under silence. Indeed, the simple PBS-washing or repeated extrusion sequences are often not efficient enough to break Van der Walls or ionic interactions. However, these can be disrupted during the in vivo circulation or along their storage. Therefore, the issues are two-fold: how can it be ensured that the desired drug quantity is actually delivered?; and can the immunogenicity or toxicity of the in vivo released ingredients and carriers be avoided? Again, even if these issues are often evoked in the field reviews, we found no published studies on these concerns.

Finally, less is known about lipid trafficking linked to exosomes. Applications connected with the recent publications on the lipid mediators implied in the inflammation regulation would benefit from more studies.

Started only ten years ago, the clinical use of exosome-like vehicles needs to address all these more technical and deep-research issues. However, considering their great potential, it is paramount to open the application field of such technologies. Indeed, most pre-clinical assays are focused on cancer therapy or gene edition. Nevertheless, exosomes are also promising candidates for vaccine development as it has been demonstrated in several vaccine studies that infected cells secrete exosomes containing multiple antigens from the pathogen.

## Figures and Tables

**Table 1:** Clinical trials running between 2011 and 2024 that involved exosomes in primary outcome measures. Source: classic.clinicaltrials.gov. Key word EXOSOME N = 171.

Pathology Type	Pathology	Study	Type	Start Date	NCT Number
Blood pathology	Sepsis	Function of circulating exosomes in sepsis-induced immunosuppression	O	15/04/2021	NCT04979767
Brain disease	Alzheimer’s disease	Safety and efficacy evaluation of allogenic adipose MSC-Exos in patients with Alzheimer’s disease	I	01/07/2020	NCT04388982
Brain disease	Parkinson’s disease	LRRK2 and other novel exosome proteins in Parkinson’s disease: biomarkers associated with Parkinson’s disease susceptibility and/or progression in exosome-proteomes derived	O	01/01/2013	NCT01860118
Cancer	Bladder cancer	Characterization of the protein profile in tumor derived exosomes from the gallbladder carcinoma patients	O	01/01/2018	NCT03581435
Cancer	Bladder cancer	Use of urinary exosome lncRNAs for preoperative diagnosis of lymphatic metastasis in patients with bladder cancer	O	01/06/2023	NCT05270174
Cancer	Bladder cancer	Use of urine exosomal RNA for accurate diagnosis of urinary tract tumors and the development of kits	O	01/06/2023	NCT06193941
Cancer	Bone cancer	Identification and characterization of miRNAs content in circulating tumor exosomes in bone metastases	O	03/12/2018	NCT03895216
Cancer	Brain cancer	Clinical relevance of detecting molecular abnormalities in glial tumor exosomes (blood sampling)	I	15/12/2023	NCT06116903
Cancer	Breast cancer	Omic technologies to track resistance to palbociclib in metastatic breast cancer: longitudinal changes in volatile organic components profile and exosomes according to response to treatment	I	08/09/2020	NCT04653740
Cancer	Breast cancer	Development of a horizontal data integration classifier for noninvasive early diagnosis of breast cancer (from different radiomics analyses on baseline mammograms and molecular analyses on peripheral blood (miRNA sequencing from exosomes) and urine	I	19/01/2021	NCT04781062
Cancer	Breast cancer	Exosome as prognostic and predictive biomarker in early breast cancer patients	I	01/05/2021	NCT05955521
Cancer	Breast cancer	Feasibility of exosome analysis in cerebrospinal fluid during the diagnostic workup of metastatic meningitis (Exo-LCR)	I	04/01/2023	NCT05286684
Cancer	Breast cancer	Study to measure the expression of the HER2-HER3 dimer in tumor and blood (exosomes) samples from patients with HER2 positive breast cancer receiving HER2 targeted therapies	O	20/12/2019	NCT04288141
Cancer	Cancer	Pilot study with the aim to quantify a stress protein in the blood and in the urine for the monitoring and early diagnosis of malignant solid tumors: concentration of HSP70 exosomes in the blood and urine	I	15/12/2015	NCT02662621
Cancer	Colon cancer	Study investigating the ability of plant exosomes to deliver curcumin to normal and colon cancer tissue	I	01/01/2011	NCT01294072
Cancer	Colorectal cancer	Contents of circulating extracellular vesicles: prognostic role of exosomes and their contents on the survival of colorectal cancer patients	O	01/07/2020	NCT04523389
Cancer	Colorectal cancer	Identification in blood sample of new diagnostic protein markers derived from circulating tumor exosomes for colorectal cancer	O	07/01/2021	NCT04394572
Cancer	Epithelial cancer Bowel disease	Plant exosomes +/− curcumin to abrogate symptoms of inflammatory bowel disease	I	01/03/2018	NCT04879810
Cancer	Gastric cancer	Circulating exosomes as potential prognostic and predictive biomarkers in advanced gastric cancer patients (“EXO-PPP study“): characterization of the molecular profile in tumor derived exosomes, correlation of plasma level and kinetics of gastric cancer derived exosomes	O	01/01/2013	NCT01779583
Cancer	Liver cancer	Early plasmatic biomarkers of tumor response in high dose hypofractionated radiotherapy. Work Package 3: Immune response (analyses of immunological parameters, description of secreted markers and nanovesicles production, verification of the presence and evolution of activation markers and quantification of secreted exosomes)	I	16/09/2015	NCT02439008
Cancer	Lung cancer	Prediction of immunotherapeutic effect of advanced non-small cell lung cancer (detection of the difference of miRNA expression profiles of exosomes in NSCLC patients before and after immunotherapy -pabolizumab, nafulizumab-)	I	08/06/2020	NCT04427475
Cancer	Lung cancer	Molecular profiling of exosomes in tumor-draining vein of early-staged lung cancer	I	21/06/2021	NCT04939324
Cancer	Lung cancer	Combined diagnosis of cancerous tissue and exosome in early lung cancer	O	20/05/2018	NCT03542253
Cancer	Lung cancer	Early diagnosis of lung cancer using blood plasma derived exosome (evaluating the possibility of distinguishing between normal and lung cancer patients through the analysis of lung cancer-specific exosomal protein)	O	09/04/2020	NCT04529915
Cancer	Lung cancer	Study of exosome EML4-ALK fusion in NSCLC clinical diagnosis and dynamic monitoring (the objective response rate of those NSCLC patients receiving ALK inhibitors treatment according to exosome ALK fusion diagnosis and fluorescence in situ hybridization examination)	O	01/08/2020	NCT04499794
Cancer	Lung cancer	Serum exosomal miRNA predicting the therapeutic efficiency in lung squamous carcinoma	O	01/04/2022	NCT05854030
Cancer	Lung cancer	Extracellular vesicles (isolate exosomes from bronchial washings) and particles as biomarkers of recurrence in non-small cell lung cancer	O	14/06/2022	NCT05424029
Cancer	Lymphoma, B-cellaggressive non-Hodgkin (B-NHL)	Exosomes and immunotherapy in non-Hodgkin B-cell lymphomas (quantification of CD20 and PDL-1 in exosomes purified from cell cultures of DLBCL human cell lines and from healthy volunteers)	I	02/07/2019	NCT03985696
Cancer	Melanoma	Analysis of circulating exosomes in plasma of melanoma patients (dosage of proteic biomarkers in circulating exosome)	O	01/03/2019	NCT05744076
Cancer	Oropharyngeal cancer	Exosome testing as a screening modality for human papillomavirus-positive oropharyngeal squamous cell carcinoma	O	25/02/2015	NCT02147418
Cancer	Ovarian cancer	Non-coding RNA in the exosome of the epithelia ovarian cancer (expression of miRNA/lncRNA, expression of micro-RNA (miRNA) and long non-coding RNA (lncRNA) compared between high grade serous ovarian carcinoma group and control group)	O	10/11/2018	NCT03738319
Cancer	Pancreatic cancer	Trial of ascorbic acid + triple therapy with nanoparticle paclitaxel protein bound + cisplatin + gemcitabine in patients with advanced stage IV metastatic pancreatic cancer	I	15/12/2017	NCT03410030
Cancer	Pancreatic cancer	Circulating extracellular exosomal small RNA as potential biomarker for human pancreatic cancer	I	01/11/2020	NCT04636788
Cancer	Pancreatic cancer	iExosomes in Treating Participants With Metastatic Pancreas Cancer With KrasG12D Mutation (Mesenchymal Stromal Cells-derived Exosomes with KRAS G12D siRNA)	I	27/01/2021	NCT03608631
Cancer	Prostate cancer	Exosomal microRNA in predicting the aggressiveness of prostate cancer in Chinese patients by comparison of the differences in microRNA expression between non-prostate cancer subjects, pathologically insignificant and significant prostate cancer patients	O	03/05/2018	NCT03911999
Cancer	Prostate cancer	ExoDx prostate evaluation in prior negative prostate biopsy setting	O	15/03/2020	NCT04357717
Cancer	Rectal cancer	Exosomes in rectal cancer (characterization of plasmatic exosomal biomarker levels in patients with locally advanced rectal cancer undergoing neoadjuvant chemoradiation therapy)	O	13/02/2018	NCT03874559
Cancer	Renal cancer	Evaluation of urinary exosomes presence from clear cell renal cell carcinoma	O	29/01/2020	NCT04053855
Cancer	Renal cancer	A companion diagnostic study to develop circulating exosomes as predictive biomarkers for the response to immunotherapy in renal cell carcinoma (blood and urine collection)	O	01/01/2023	NCT05705583
Cancer	Sarcoma	Study of blood exosomes in monitoring patients with sarcoma (EXOSARC)	O	19/11/2018	NCT03800121
Cancer	Systemic autoimmune diseases	Urine miRNAs-exosomes to identify biomarkers for lupus nephritis	O	02/08/2020	NCT04894695
Chronic disease	Diabetes type 2	Determine the levels of circulating extracellular vesicles released by human islets of langerhans in plasma	O	01/12/2016	NCT03106246
Chronic disease	Diabetes type 2	Mechanisms behind severe insulin resistance during pregnancy in women with glucose metabolic disorders (SIR-MET) maternal hormonal, inflammatory and metabolic markers in the blood, as well as the level, content and bioactivity of exosomes are studied	O	01/05/2021	NCT04924504
Chronic disease	Diabetic retinopathy	Role of the serum exosomal miRNA (sequencing) in diabetic retinopathy	O	01/07/2018	NCT03264976
Chronic disease	Diabetic retinopathy	Study on exosome changes from plasma, atrial fluid and vitreous fluid in patients with proliferative diabetic retinopathy by proteomic analysis of proteins	O	01/01/2024	NCT06198543
Chronic disease	Diabetic retinopathy	Proteomic study of plasma exosomes in patients with diabetic retinopathy	O	02/01/2024	NCT06188013
Chronic disease	Hypertension	New biomarkers and difficult-to-treat hypertension; concentrations and variabilities of urinary exosomal sodium channels and plasma angiotensins in patients with difficult-to-treat arterial hypertension	O	01/05/2016	NCT03034265
Endothelial pathology	Endothelial dysfunction	Circulating exosomes and endothelial dysfunction in patients with obstructive sleep apneas hypopneas syndrome: miRNA contained in exosomes, comparison of exosome content between obese-OSA patients with endothelial dysfunction and without	I	14/01/2021	NCT04459182
Genetic disase	Dystrophic epidermolysis bullosa	Study to assess the effectiveness and safety of AGLE-102, an allogeneic derived MSC EVs product derived from normal donor on lesions in subjects with dystrophic epidermolysis bullosa	I	01/01/2024	NCT04173650
Heart pathology	Cardiac remodeling	Exosome as integrative tool for prognostic stratification of adverse cardiac remodeling in stemi patients: the MIRACLE Study (verify whether the profile of circulating plasma exosomes reflects cardiovascular magnetic resonance in acute ST-segment–elevation myocardial infarction)	O	03/05/2023	NCT06070974
Heart pathology	Atrial fibrillation	Role of exosomes derived from epicardial fat in atrial fibrillation: analysis of the differences in the quantity of exosomes derived epicardial fat biopsy in patients with and without atrial fibrillation.	I	21/01/2018	NCT03478410
Infectious disease	COVID-19	Intravenous infusion of CAP-1002 (cardiosphere-derived cells) in patients with COVID-19	I	15/11/2020	NCT04623671
Infectious disease	COVID-19	The use of exosomes, delivered intravenously-ARDOXSO™, for the treatment of acute respiratory distress syndrome or novel coronavirus pneumonia caused by COVID-19	I	2023-09	NCT04798716
Infectious disease	HIV	Inflammation, NK cells, antisense protein and exosomes, and correlation with immune response during HIV infection	I	22/04/2022	NCT05243381
Respiratory pathology	Acute respiratory distress syndrome	Screening of differential miRNAs of inflammatory exosomes in plasma and alveolar lavage fluid of patients with sepsis complicated with acute respiratory distress syndrome	O	25/07/2022	NCT05476029
Respiratory pathology	Obstructive sleep apnea syndrome	Exosomes implication in PD1-PD-L1 activation in obstructive sleep apnea syndrome	O	03/03/2019	NCT03811600
Respiratory pathology	Acute respiratory distress syndrome	Safety and efficacy of EXO-CD24 in preventing clinical deterioration in patients with mild–moderate acute respiratory distress syndrome	I	04/07/2023	NCT05947747
Respiratory pathology	Respiratory dysfunction	Blood circulating microRNAs as biomarkers of respiratory dysfunction in patients with refractory epilepsy	I	16/03/2018	NCT03419000
Systemic autoimmune diseases	Psoriasis	Safety and tolerability study of MSC exosome ointment	I	08/03/2022	NCT05523011

I: interventional, O: observational.

## Abbreviations

cNPs—cellular membrane coated nanoparticles; Cur—curcumin; DDS—drug-delivery system; Dox—doxorubicin; EGF—epidermal growth factor; ESCRT—endosomal sorting complex required for transport; EVs—extracellular vesicles; HNVs—hybrid nanovesicles; HE—hybrid exosomes; LNPs—lipid nanoparticles; MSC—mesenchymal stem cells; miRNA—microRNA; nanoEV—nanometric extracellular vesicles; Ptx—paclitaxel; siRNA—small interfering RNA.
